# Has the Data Efflux Regarding the Promising Outcome Following Injection of Deflux Changed the Management of Adult Vesicoureteral Reflux?

**DOI:** 10.1155/2008/361324

**Published:** 2009-03-10

**Authors:** D. E. Zilberman, Y. Mor

**Affiliations:** Department of Urology, The Chaim Sheba Medical Center, Sackler School of Medicine, Tel-Aviv University, Tel-Hashomer, Ramat-Gan 52621, Israel

## Abstract

Primary vesicoureteral reflux (VUR), traditionally considered a problem of childhood, can also be detected during adulthood. However, while the concept regarding the therapeutic management of VUR in children has undergone revolutionary changes, moving from surgical to conservative approach, the optimal therapeutic approach in adult reflux is poorly addressed and is still unknown. Herein, we review clinical and therapeutic approaches of VUR in pediatric population as published throughout the years. With the introduction of Deflux injection as a minimally invasive procedure, we identify a beginning of a new trend that further extends the indications for endoscopic injections, including its introduction to adult patients as well.

## 1. INTRODUCTION

Primary vesicoureteral reflux (VUR), traditionally considered a problem of childhood, can also be detected during adulthood [1]. However, while the concept regarding the therapeutic management of VUR in children has undergone revolutionary changes, moving from surgical to conservative approach, based upon solid prospective data, the optimal therapeutic approach in adult reflux is poorly addressed in the literature and is still unknown. The current therapeutic strategy for management of reflux has drawn its inspiration from three important large prospective studies dealing with the management of VUR [2–4]. The most comprehensive was the International Reflux Study (IRS), where 452 patients in Europe and USA were randomly allocated to medical and surgical arms. In 5 years follow-up, the same incidence of urinary tract infection (UTI) was seen in both arms (38%), though surgery was more effective in preventing pyelonephritis (PN) (21% versus 10%). However, in 10 years follow-up, clinical findings did not support the surgical attitude as there was no significant difference in renal growth comparing both arms, and there was no support to the view that the outcome of renal function is improved by surgical correction of VUR in children with bilateral disease. These studies had led to the publication of the clinical guidelines for the management of VUR in children, both by the American Urological Association (AUA) [5] and the European Association of Urology (EAU) [6].

In general, conservative attitude is currently the mainstay, and surgical intervention takes its place in the more severe conditions. While those observations are extremly important in children, they are irrelevant for adults as factors such as the natural history of the disease, the associated risks such as infections or scars are completely different. Unfortunately, with regard to VUR in adults, review of the literature reveals only few retrospective studies, some of them biased with conflicting results.

## 2. PREVALENCE OF VUR IN ADULTS

In the general pediatric population, the prevalence of VUR is around 1-2% with higher rates in siblings (30%) and in children with acute PN (25–40%) [5].

As the rates of disappearance of VUR in children are as high as 71% and can occur at any age, in infancy or at puberty [7], the actual prevalence in adults is still unclear. Baker and coworkers [8] found an incidence of 26.4% of VUR in children but only 5.2% in adults, each group suspected of “having infravesical obstruction”.

Similarly, Choi et al. [9] studied 86 adult women suffering from uncomplicated PN with voiding cystourethrogram (VCUG) performed on the 3rd and the 7th days of antibiotic treatment, and only in 2 cases (2.3%), VUR was demonstrated.

Pinthus et al. [10] explored the same situation in 47 women who presented with acute PN. Early indirect cystography revealed VUR in 28%, while VCUG performed later found VUR in only 3 patients (9%). In accordance, while the resolution rates of reflux in children are rather predicted, it seems that the possibility of resolution even past puberty still exists, yet the chance for resolution is probably much less than in infants [11].

## 3. PRESENTATION

The most common initial symptoms and findings that can lead to the diagnosis of VUR are UTI, asymptomatic bacteriuria, proteinuria, renal failure, and hypertension [12]. Köhler et al. followed after 115 adult patients and found UTIs in 87%, hypertension in 34%, renal calculi in 18%, and back pain in 42% [12]. Vice versa, reflux nephropathy may be clinically latent as the prevalence of reflux in patients with incidentally diagnosed adult hypertension exceeds to 19%, without any apparent renal parenchymal or renovascular involvement [13]. However, the correlation between presence of VUR and various clinical presentations cannot be made that easily as different, and somehow confounding observations were published in other studies and in some patients the reflux may be even completely asymptomatic [11].

## 4. THE CURRENT APPROACH FOR MANAGEMENT OF ADULT VUR

So far, although no evidence-based recommendations are available in the literature, the last AUA update (1998) [11] for the treatment of VUR in adolescents and adults recommends the following.

- No medical management is needed in VUR grade 1-2 and no history of UTI.
- Medical management with lifelong antibacterial prophylaxis should be considered in the cases of low-grade VUR, shortened life expectancy, and poor surgical risk.
- Surgery is indicated in VUR grade 3 or higher, history of recurrent PN, and evidence of nephron loss.

When conservative treatment is the mainstay, progressive renal damage and caliceal scarring should be expected [14]. Reimplantation, when performed, does not improve hypertension or renal failure, but it rather stops the anticipated progressive deterioration [10, 15].

Similar conclusions were coming up from Köhler and Guthman's studies [16, 17]. In the first study [16], surgical treatment (e.g., ureteral reimplantation) did not alter the frequency of lower UTI, though it significantly decreased the frequency of PN. Yet, the surgical option according to the author should be considered only when conservative treatment failed and not with the aim of arresting renal functional deterioration. However, the second study [17] had expanded the indications for surgical treatment also for asymptomatic women in childbearing age “in whom pyelonephritis of pregnancy would pose a major risk to the fetus and mother”, without supporting evidences. Ever since, most clinicians recommend that surgical correction of VUR should be accomplished before pregnancy in women at childbearing age or even earlier in girls with reflux that persists beyond puberty.

This recommendation is based upon the fact that history of VUR is known to increase morbidity during pregnancy including the risk of preeclampsia, obstetric interventions, and fetal loss. Women with hypertension and an element of renal failure are particularly at risk, though surgical correction does not prevent complications but rather decreases their frequency [18]. It should be also remembered that reimplantation in adults is more difficult with lower success rates compared to infants. This can be attributed to difficult bladder exposition, increased vascularity around the ureter and in the retrovesical space, and increased body mass [11].

Altogether, VUR in adults is still a very controversial subject, and throughout the years, the pendulum had moved from surgical to a more conservative treatment and again to surgical treatment in certain and severe cases.

## 5. ENDOSCOPIC CORRECTION OF VUR

During the last seven years, we are witnessing an increasing number of studies published in the English literature, discussing the safety and efficacy of endoscopic injection of bulking materials for the correction of ureteric reflux. The need for alternative treatment aroused as a result of the significant disadvantages of both reimplantation and antibiotic prophylaxis. Reimplantation in the pediatric population carries significant cost, morbidity, and inpatient hospital stay, while antibiotic prophylaxis requires annual imaging which is expensive, invasive, and often requires sedation [19].

The first report on endoscopic injection of polytetrafluoroethylene (Teflon) in pigs came from Puri and O'Donnell in 1984 [20]. Later on, long-term results were published covering 8332 children and 12251 refluxing ureters [21], and the final conclusion was that “polytetrafluoroethylene injection is a simple, safe and effective outpatient procedure for treating all grades of vesicoureteral reflux.” Chertin and Puri [22] supported their own conclusion by reporting long-term (e.g., six years) follow-up among 258 patients with primary VUR who were treated by polytetrafluoroethylene injection. They reported overall success rates of 77% following one injection, 13.5% success rate following two injections, 2.6% following three injections, and 0.5% following four injections. Yet, the initial enthusiasm from PTFE has disappeared following the observation that small particles can be injected directly into capillaries and embolize to distant organs, causing the FDA to withdraw PTFE from the United States market [23]. Remaining with the impressive results of the endoscopic injection for treating vesicoureteric reflux, alternative materials took the PTFE place in order to keep its momentum of success, while keeping complication rates as low as possible. At present, the most popular injectable material is dextranomer/hyaluronic acid (Dx/HA) copolymer (Deflux) which is FDA approved. This is an organic substance comprising 80–250 *μ*m microspheres which are nonallergic, nonimmunogenic, and have no potential for malignant transformation [24, 25]. The large size of the microspheres prevents them from migrating outside the urinary bladder and they do not tend to form granulomas or induce calcifications [24].

Injection procedure consists of injecting the bulking material through direct inspection and under general anesthesia. Approximately, 1 mL of Deflux is submucosally injected through a special needle, which is inserted 2-3 mm below the affected ureteral orifice at the 6 o'clock position [25, 26]. The needle is slowly withdrawn as a “volcanic bulge” starts to create [25]. Overall procedure length does not exceed 30 minutes [26, 27], and the patient is discharged home the same day [19, 23, 27, 28] or the following day [24].

Overall complication rate is very low, and the procedure is considered very safe and effective. UTI, flank pain, postoperative ureteral obstruction, retrograde tracking of Deflux, intravesical extravasation of Deflux, and new contralateral VUR were reported in only few percents (0.6–4.5%) [23, 28, 29].

Success rates, on the other hand, are high and reported in various series with regard to the number of injections required to cure VUR and to the original reflux grade. Following a single injection, the reported success rates in pediatric population vary between 72–86%, following two injections—between 12-13% and following three injections—between 1-2% [23, 25, 27, 28]. Overall, cure rates reached 82–100% for grade 1 reflux, 82–88% for grade 2, 73–87% for grade 3, 64–73% for grade 4, and 50% for grade 5 [19, 23, 28]. Kirsch et al. [23] describe the lowest success rates (60%) in the first twenty cases, meaning that a reasonable learning curve exists for this certain procedure.

## 6. THE EVOLVEMENT OF THE THERAPEUTIC APPROACH IN ADULTS

All the advantages mentioned with regard to endoscopic treatment for VUR in children can definitely change the concept regarding the treatment of VUR in adults. Arguments such as high success rates, very short hospital stay, absence of significant postoperative complications, safety of injectable materials, and low cost compared to the cost of long-term antibiotic prophylactic treatment which have been raised in discussions regarding children [30–32], are also valid concerning adults. Understandably, this can widen the circle of patients treated with bulking agents to include also adults. However, in oppose to increasing reports regarding using this technique in the pediatric age group, the reported experience in adults with Deflux is very limited [19, 29, 33]. Those reports usually describe the outcome of injection of various substances in series composed of mixed populations, including children and adults. Although there is usually no specific reference to the adult subgroup, it is obvious that there has been some experience with injection of cross-linked bovine collagen [34] or STING [35, 36] in adults, with cure rates as high as 86% following the injection of polymethylsiloxan in adult women or 70% following the first injection of Teflon in adults [36].

Nowadays, in the “Deflux era”, review of the literature as well as presentations in urological conferences can identify the beginning of a new trend that further extends the indications for endoscopic injections, including its introduction to adult patients as well. Some current pediatric reports [19, 29] include in their series some adult patients as old as 22 years. Unfortunately, they do not specify this unique population in terms of number of individuals, sex, indications for Deflux injection, age at injection, follow-up length, complications, and success rates. However, we can assume that both groups were stunned by their impressive success rates, and taking into consideration that endoscopic treatment for VUR is “self and efficacious with low-complication rate” [19], they decided to offer it to certain individuals that traditionally were excluded from any definitive treatment. Enthusiasm from the introduction of Deflux injection for adult population was also expressed by Kirsch [37] who achieved success rates of 90 and 95% after one or two treatments in 22 patients ranging in age between 13–71 years.

In summary, the efflux of data regarding the safety and the promising results of Deflux endoscopic correction of VUR in children will certainly change the management of VUR in adults which unfortunately has been poorly addressed and controversial till recently. In similar with the shifting therapeutic policy of adult ureteropelvic junction obstruction following the arrival of the endourological era [38], one can likewise anticipate that “it would be unethical to refrain from treating” [30] adult patients diagnosed with VUR. Furthermore, as the procedure is safe, less invasive, highly successful, and can be repeated, we foresee that a more active strategy, namely, early endoscopic correction, will become the new gold standard of treatment of adult VUR, and we hope that this shift of policy will be clearly reflected in the coming updated clinical urological guidelines for management of VUR.
